# Dynamic Tracking of *In Vivo* Receptor Availability in Tumor Using Paired-Agent Imaging

**DOI:** 10.1021/acs.molpharmaceut.5c00060

**Published:** 2025-05-14

**Authors:** Yichen Feng, Xiaochun Xu, Cody C. Rounds, Sassan Hodge, Kenneth M. Tichauer, Kimberley S. Samkoe

**Affiliations:** † Geisel School of Medicine, 3728 Dartmouth College, 1 Rope Ferry Road, Hanover, New Hampshire 03755, United States; ‡ Thayer School of Engineering, Dartmouth College, 15 Thayer Drive, Hanover, New Hampshire 03755, United States; § Biomedical Engineering, 2455 Illinois Institute of Technology, 3255 S Dearborn Street, Chicago, Illinois 60616, United States

**Keywords:** receptor availability, paired-agent imaging, fluorescence molecular imaging, ABY-029, epidermal growth factor receptor

## Abstract

Quantitative assessment of receptor availability (RA) provides valuable insight into therapeutic outcomes in drug development and clinical practice. Here, paired-agent imaging (PAI) is used to dynamically track the availability of the epidermal growth factor receptor (EGFR) in response to *in vivo* ligand or inhibitor binding in individual mice with head and neck cancer (HNC). Naïve (*n* = 3) or xenograft HNC tumor-bearing (*n* = 21) mice were coadministered 0.15, 0.3, or 0.9 nmol ABY-029, and 2.5 nmol of IRDye 700DX. Fluorescence images were acquired for 300 min and then for an additional 60 min after administration of Z03115 (test group), human EGF (positive control), or PBS (vehicle control). Kinetic fluorescence and PAI curves were evaluated to determine the effects of the ABY-029 dose and EGFR blocking on tumor RA estimation. Nonquantifiable increases in ABY-029 fluorescence in tumor and muscle were observed after *in vivo* blocking, while PAI produced the expected decrease in RA. No statistically significant difference in preblocking RA was observed with different doses of ABY-029. RA decreased in response to blocking in positive control and test group animals, while the vehicle group exhibited no significant change in RA. This study demonstrated that RA can be monitored dynamically in individual animals using PAI regardless of imaging agent dose, while fluorescence from the receptor-targeted imaging agent alone could not. These results demonstrate PAI as a simple imaging strategy that could allow dose optimization in pharmaceutical development and patient-specific dosing for molecular therapeutics.

## Graphical abstract

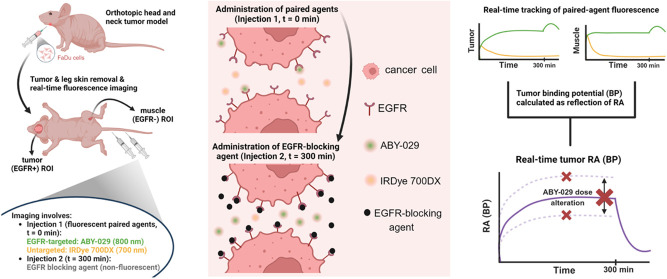

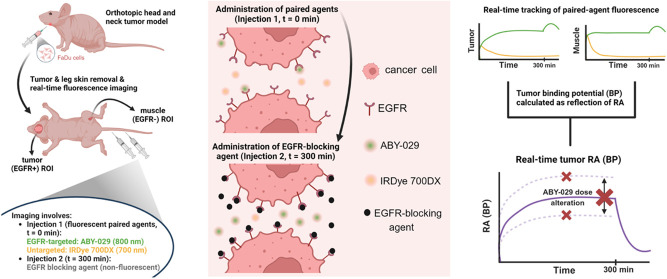

## Introduction

In the past few decades, advancements in technology have led to numerous achievements in cancer therapeutics. However, the process of clinical drug development for new therapeutics remains long (∼10–15 years) and expensive (>$1–2 billion).^1^ Despite tremendous efforts, cancer drugs still fail at a rate of over 90% largely due to high toxicity and lack of clinical efficacy;^2^ therefore, careful dose selection remains a vital step in clinical trial design. Common approaches for dose selection require evaluation of the dose–response relationship by drug pharmacokinetic (PK) and pharmacodynamic (PD) features.^3^ Although PK parameters are commonly derived *in vivo*, PD parameters are typically derived *in vitro* or *ex vivo*, owing to the complexity of physiological systems and heterogeneity of cancer. Receptor availability (RA), which represents the concentration of receptors available for drug binding, is a PD parameter that could help bridge the understanding of heterogeneous dose–response effects in individual patients;^3,4^ however, individual dose optimization requires real-time, *in vivo* therapeutic drug monitoring.^5,6^ Therefore, precise RA assessment in individual subjects could have great impacts on cancer drug development, therapeutic outcome assessment, and post-therapeutic patient monitoring.^5,7^

Molecular imaging has been viewed as a powerful technology to evaluate drug-target engagement *in vivo*.^2,4,8,9^ Modalities including fluorescence imaging, positron emission tomography (PET), and single photon emission tomography (SPECT) employ receptor-targeted imaging agents to allow the acquisition of physiological information at the molecular scale.^10–12^ Competitive receptor binding between imaging agents and drug molecules to assess drug RA has been demonstrated extensively in the central nervous system (CNS) using PET and SPECT.^13–15^ However, the advancement of tumor RA measurement remains slow, as the delivery of imaging agents to tumors is greatly affected by abnormal and complex hemodynamic and physiologic factors, including leaky vasculature, deficient lymphatic drainage, heterogeneity of receptor expression, and nonspecific tissue uptake.^16–19^ Consequently, direct quantification of RA from imaging agent signal intensity in tumors is hindered by complex signals arising from receptor-bound agents and unbound agents remaining in the tumor and blood.^20^ For the same reason, common approaches to separate signals from nonspecific tissue uptake in the brain, such as arterial blood sampling or quantifying nonreceptor expressing region (“reference tissue”), are not appropriate for tumors.^21–24^ Moreover, the clinical application of kinetic analysis in nuclear medicine remains at the perfusion or filtration level clinically in cardiology and nephrology; RA parametric analysis is still under research-only status.^25,26^

Certain methodologies to measure cancer drug RA exist with mixed strengths and weaknesses. Flow cytometry, for example, has been used extensively for drug assessment; but its usage is limited to circulating cells due to complex tumor structure and hemodynamics. *Ex vivo* evaluation of RA for solid tumors lacks temporal information on drug distribution and interactions; and has demonstrated large data variation originating from sample preparation.^3,7^

Efforts have been made to assess RA for drug compounds through *in vivo* measurements, including the usage of molecular imaging.^19,27^ Dubach et al. employed a method combining competitive binding with fluorescently labeled companion imaging probes (CIP) for indirect measurement of drug compound engagement, achieving high spatial resolution *in vivo* detection of drug–receptor engagement.^19^ However, this approach necessitates the use of a dorsal window chamber for microscopic single-cell imaging, limiting tumor growth to a few cubic millimeters and simplifying the tumor model’s complexity (e.g., vascular and microenvironment). Moreover, the topical administration of drugs and probes restricts its applicability in clinical settings. Tang et al. developed a bioluminescence resonance energy transfer (BRET)-based approach for precise *in vivo* quantification of RO in a mouse tumor model, offering an enhanced signal-to-noise ratio.^27^ However, this approach necessitates linking both the drug and the receptor with BRET components to measure bioluminescence. Such modification of the drug requires additional evaluation to ensure comparable *in vivo* pharmacokinetics to the unmodified version. Moreover, the need to transfect label-modified receptors into tumor tissue limits the method’s scalability across multiple drug candidates and patient-derived tissues. Other *in vivo* approaches apply systemic administration of receptor-specific competitive probes to measure unbound receptors.^28,29^ With no requirement for tissue or drug modification, these assays are more promising to guide individualized dosing. However, due to nonspecific tissue uptake, probe signals detected do not directly reflect receptor binding.^24,30^ Therefore, there remains a need for a method capable of assessing RA in tumors without requiring receptor premodification.

We have previously reported a near-infrared, paired-agent imaging (PAI) strategy designed to compensate for the nonspecific uptake of imaging agents in the tumor.^30,31^ PAI employs simultaneous administration of two imaging agents: a receptor-targeted agent and an untargeted agent devoid of receptor specificity. The agents are coadministered at trace levels (*i.e*., results in <5% of receptor being bound) and fluoresce at different wavelengths, allowing for signal separation and estimation of “binding potential” (BP) using a PAI version of the simplified reference tissue model (or an instantaneous ratio of the targeted and control signals).^24,30^ BP is equal to the product of the concentration of receptor available for ligand binding and the affinity of the targeted imaging agent to the receptor.^24,30,32^ Nuances in PK between targeted and untargeted agents can be accounted for by deconvolving the uptake of the tracers in tissues devoid of targeted agent binding.^33,34^ In previous PAI studies, tumor BP was used to report the change in RA between control and drug-treated groups at a single time point after treatment.^30,35^ On the other hand, PET and SPECT have been used to dynamically track drug RA using BP in the brain.^36,37^ However, dynamic tumor RA tracking in individuals before and after administration of an inhibitor or ligand using PAI has not been assessed.

We hypothesized that near-infrared PAI is capable of dynamic tracking of tumor RA in response to receptor blocking in an individual subject. To test this hypothesis, kinetic fluorescence imaging was performed in mice bearing orthotopic epidermal growth factor receptor (EGFR)-positive head and neck cancer (HNC) xenografts. Real-time imaging of both an EGFR-targeted agent, ABY-029, and a suitable control agent, IRDye 700DX,^38^ was performed until steady-state ABY-029-EGFR binding, followed by administration of a saturating dose of a nonfluorescent, EGFR-targeted agent. It was anticipated that both ABY-029 fluorescence and BP would decrease in the tumor after blocking, but BP would more accurately predict the observed change in RA. Furthermore, to demonstrate the independence of BP from targeted agent dosage, three different doses of ABY-029 were paired with IRDye 700DX in this study, while the blocking agent dose was held constant. If successful, the ability to quantitatively track *in vivo* RA in individual subjects has far-reaching implications for both drug discovery and individualized patient therapies clinically.

## Materials and Methods

### Cell Lines and Culture Methods

FaDu, a human squamous cell carcinoma cell line, was purchased from ATCC (Manassas, VA) and cultured in Dulbecco’s modified Eagle’s medium (DMEM) with 10% fetal bovine serum and 1% penicillin–streptomycin.

### Imaging Agents

The EGFR targeted agent, ABY-029, was synthesized from the conjugation of Z03115-Cys-trifluoroacetate salt (Z03115, Affibody Medical, Sweden and Bachem AG, Switzerland) to IRDye 800CW maleimide (LI-COR Biosciences, Inc., Lincoln, NE) under Good Laboratory Practice (GLP), as described previously.^39,40^ The untargeted agent, IRDye 700DX NHS ester (LI-COR Biosciences, Inc.), was converted to its carboxylate form by dissolving the dye in phosphate-buffered saline (PBS, pH = 8.5) and stirring at room temperature for 5 h, as per instructions from the manufacturer. Subsequent dilutions of both agents were performed in PBS at a pH of 7.0 for administration.

### Nonfluorescent Blocking Agents

Z03115 (unlabeled ABY-029) was utilized as the test agent, while recombinant human epidermal growth factor (hEGF, Millipore, Temecula, CA) was applied as a positive control. Phosphate-buffered saline (PBS, Corning, NY) was employed as the vehicle control agent.

### Mouse Xenograft Model

All animal procedures were conducted according to a protocol approved by the Dartmouth Institutional Animal Care and Use Committee (IACUC) certified by the NIH-OLAW and AAALAC guidelines. Female, athymic nude mice, 6–8 weeks of age (*N* = 24), were purchased from Jackson Laboratory (Bar Harbor, ME). Twenty-one mice were implanted with 1 × 10^6^ FaDu cells in 50 μL of culture medium by transcervical injection into the base of the tongue. The mice were imaged when the tumors reached 100 mm^3^ (∼2 weeks after implantation). Three naïve (no-tumor control) mice were also included.

### Animal Groups

As described in Figure 1, mice with xenograft tumors (*N* = 21) were separated into positive control (*n* = 9), test (*n* = 9), and vehicle control groups (*n* = 3). The three naïve mice formed the “naïve test control” group. ABY-029 was administered at 0.15, 0.3, or 0.9 nmol per animal for the positive control and test groups (*n* = 3 per dose) and 0.9 nmol for the vehicle and naïve test control groups. All doses of ABY-029 were mixed with 2.5 nmol of IRDye 700DX in 200 μL of PBS (“paired-agent solution”) and injected into the tail vein. Blocking agents were administered via tail vein injection in 100 μL of PBS at 300 min after the paired-agent solution injection as follows: PBS (vehicle), 31.25 nmol hEGF (positive control), and 31.25 nmol Z03115 (test and naïve test control).

**Figure 1:**
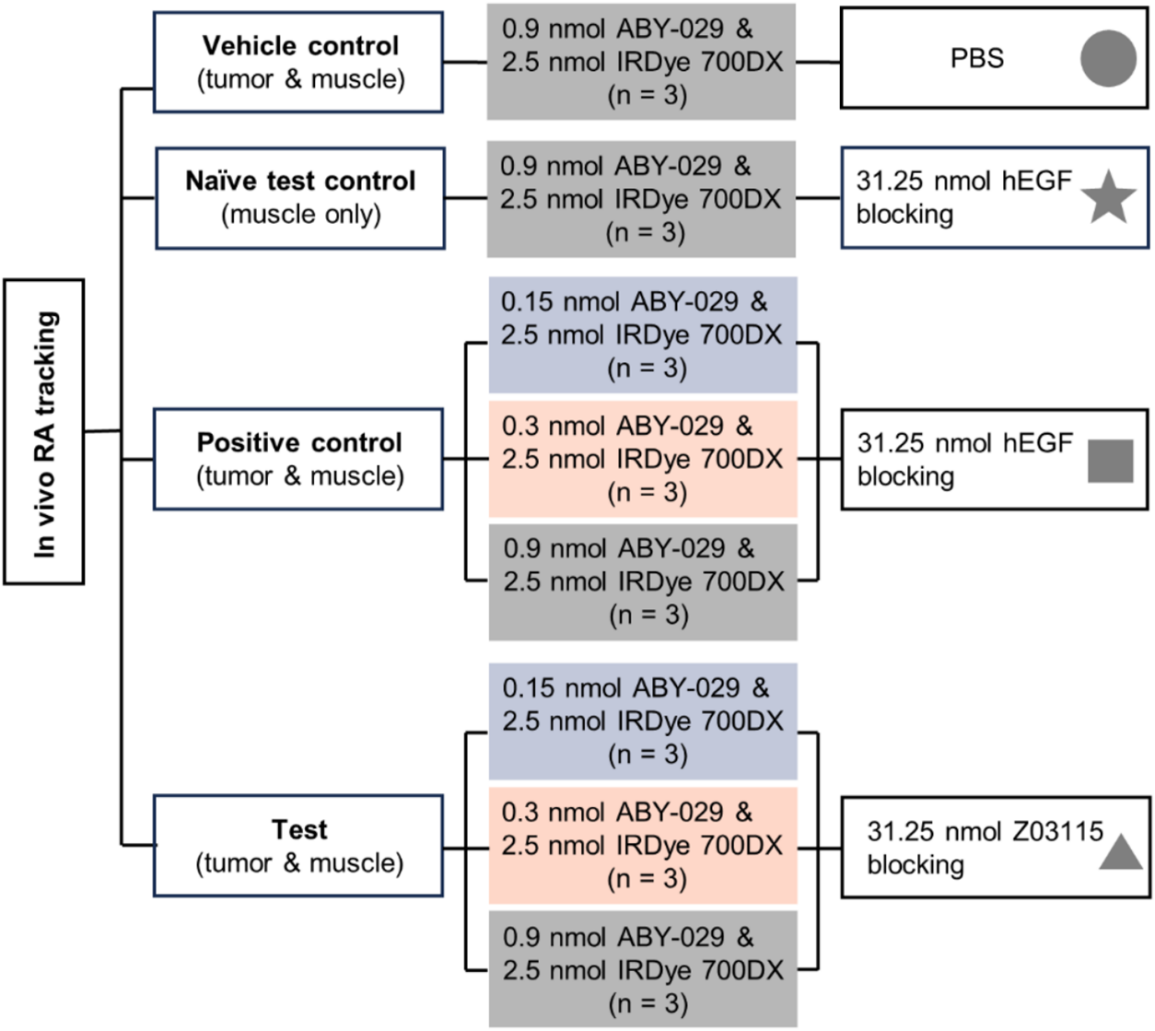
Schematic illustration of animal groups and dosing strategies.

### Fluorescence Imaging

Mice were anesthetized for imaging using 0.9–2% isoflurane in 1 L/min of oxygen. The skin covering the tumor and the leg muscle was removed, and the exposed areas were covered with plastic wrap to retain moisture. A similar procedure was applied to naïve mice, with the exception that both leg muscles were exposed for imaging. Mice were placed on the heated bed of the Pearl Impulse (LI-COR Biosciences, Inc.) and covered with black aluminum foil (ThorLabs, Newton, NJ) to reduce the contribution of fluorescence from internal organs while leaving the tumor and muscle areas exposed. For each time point, image sets were collected in the white-light, 700 nm (IRDye 700DX), and 800 nm (ABY-029) channels at 84 μm resolution. An image set was collected before and immediately after paired-agent injection and then collected every 2 min for the first 30–60 min followed by every 6 min until 300 min. At this time point, there was an apparent plateau in the fluorescence kinetic curves, indicating that the system was near steady-state. At 300 min, 100 μL of blocking or vehicle solution was administered, and image set collections were continued every 2 min for 60 min. Animals were euthanized after 360 min of imaging. Raw fluorescence images were exported as 22-bit grayscale tiff images for analysis.

### Image Analysis and Receptor Availability Calculation

Time series fluorescence images for each mouse were analyzed using FIJI.^41^ Regions-of-interest (ROI) for the tumor and muscle were drawn on the white light channel of the autofluorescence image. The ROIs were transferred to both 700- and 800 nm images, and tissue-specific fluorescence of each channel and time point was recorded as the mean fluorescence intensity (MFI) of the ROI. For each time series MFI set, background subtraction was performed using the autofluorescence values, followed by normalization using MFIs of 6–16 min postpaired-agent injection. Single-time point (STP) BPs were calculated for each ROI over the 360 min of imaging [BP­(*t*)] for all mice^35,42^ using the following equation

1 $$\begin{array}{l} \mathrm{BP}\left(t\right)=\frac{I_{\mathrm{tt}}\left(t\right)}{I_{\mathrm{tu}}^{\mathrm{CF}}\left(t\right)}-1 \end{array}$$

where *I*_tt_(*t*) represents the time-dependent ABY-029 fluorescence intensity in the tumor and *I*_tu_^CF^(*t*) represents the time-dependent IRDye 700DX fluorescence intensity in the tumor, modified by a correction function that accounts for PK differences between the two agents. Two methods were used to calculate the STP BP correction function: the “deconvolution” and “ratiometric” methods. The deconvolution approach was utilized in previous studies to correct for differences in the arterial input function of the targeted and untargeted agents.^31,33^ Calculation of the deconvolution correction function, *g*(*t*), for STP BP (BP_deconv_) was extracted from

$$\begin{array}{l} {I_{tu}^{CF}\left(t\right)}_{deconv}=I_{tu}\left(t\right)*g\left(t\right) \end{array}$$ 2

where *I*_tu_(*t*) represents the time-dependent IRDye 700DX fluorescence intensity in the tumor, ∗ is a convolution operator, and *g*(*t*) was calculated by deconvolving the time-dependent autofluorescence-subtracted ABY-029 and IRDye 700DX fluorescence in the muscle [*I*_mt_(*t*) and *I*_mu_(*t*), respectively] from

$$\begin{array}{l} I_{mt}\left(t\right)=I_{mu}\left(t\right) \end{array}*g\left(t\right)$$ 3

Deconvolution performed to calculate *g*(*t*) from eq 3 was based on general singular value decomposition (SVD) and Tikhonov regularization, with the regularization parameter being 0.01.^33^

For the ratiometric approach, STP BP at each time point (BP_ratio_) was calculated using the correction factor^30^

4 $$\begin{array}{l} {I_{\mathrm{tu}}^{\mathrm{CF}}\left(t\right)}_{\mathrm{ratio}}=I_{\mathrm{tu}}\left(t\right)\times \frac{I_{\mathrm{mt}}\left(t\right)}{I_{\mathrm{mu}}\left(t\right)} \end{array}$$

### Profiling of *In Vivo* Fluorescence and Receptor Availability

Pre- and postblocking RAs were characterized for each mouse, and methods of calculation are summarized in Table 1. Briefly, all preblocking fluorescence, BP_deconv_, and BP_ratio_ were calculated by averaging the corresponding values within the 240–300 min time range. All postblocking ABY-029 fluorescence values were determined as the maximum values of fitted second-order polynomial curves in the 300–360 min range. All postblocking values of BP_deconv_ and BP_ratio_ were calculated as the minimum values of fitted second-order polynomial curves in the 300–360 min range. Note that there was no tumor fluorescence or BP for the naïve test control mice. All second-order polynomial fittings were performed for individual animals by using the Curve Fitting Toolbox of MATLAB version R2022b.

**Table 1:** Methods to Calculate Pre- and Post-Blocking ABY-029 Fluorescence and BP^a^

parameter	tissue	time range	vehicle	positive control	test	naïve test control
fluorescence (AU)	tumor	preblocking	*I̅*_tt_	*I̅*_tt_	*I̅*_tt_	NA
		postblocking	*I̅*_tt_	*max*[p*oly*2*fit*(*I*_*tt*_)]	*max*[*poly*2*fit*(*I*_*tt*_)]	NA
	muscle	preblocking	*I̅*_mt_	*I̅*_mt_	*I̅*_mt_	*I̅*_mt_
		postblocking	*I̅*_mt_	*max*[*poly*2*fit*(*I*_*mt*_)]	*max*[*poly*2*fit*(*I*_*mt*_)]	*max*[*poly*2*fit*(*I*_mt_)]
BP	tumor	preblocking	$\overset{®}{\mathrm{BP}}$	$\overset{®}{\mathrm{BP}}$	$\overset{®}{\mathrm{BP}}$	NA
		postblocking	$\overset{®}{\mathrm{BP}}$	*min*[*poly*2*fit*(*BP*)]	*min*[*poly*2*fit*(*BP*)]	NA

a AU: arbitrary unit, BP: binding potential, NA: not applicable.

### Statistical Analyses

All graphing and statistical analyses were performed using GraphPad Prism version 10.1 (GraphPad Software, San Diego, CA). One-way analysis of variance (ANOVA) with Tukey’s test was performed to compare preblocking BP among all animals given 0.15, 0.3, or 0.9 nmol ABY-029. Based on the preassumptions of postblocking values of ABY-029 fluorescence and BP being no larger than preblocking values, one-tailed, two-way repeated-measures (RM) ANOVA with *in vivo* blocking as a repeated-measures factor and the animal group as a nonrepeated factor was employed to compare group-wise responses to *in vivo* blocking of tumor ABY-029, muscle ABY-029 fluorescence, and BP. Additional one-tailed, RM ANOVA with *in vivo* blocking as a repeated-measures factor and the treatment group as a nonrepeated factor was employed, comparing BP among the three treatment groups: vehicle, positive control, and test. Further pre- vs postblocking comparisons within each animal group or treatment group were performed using Tukey’s test. Statistical significance was based on *p* < 0.05. All data were presented as mean ± standard deviation (SD).

## Results

### Kinetic Fluorescence of Paired Agents

Kinetic fluorescence curves of ABY-029 (800 nm) and IRDye 700DX (700 nm) in tumor and muscle (EGFR-negative control tissue) are plotted in Figure 2, categorized by the animal group and tissue type. During the preblocking period, the fluorescence of IRDye 700DX in both tumor and muscle tissues remained consistent across all animal groups, exhibiting a rapid increase followed by a gradual decrease that was more pronounced in muscle (Figure 2B,D,F and 2A,C,E,G, dashed curves). Group-wise consistency in kinetic ABY-029 fluorescence curves was also observed, characterized by initially fast and then gradual rise to plateau in tumor (Figure 2B,D,F, solid curves) and a rapid increase to steady-state in muscle (Figure 2A,C,E,G, solid curves). *In vivo* receptor blocking started at 300 min, with mice xenografts receiving either Z03115 (test group), PBS (vehicle), or hEGF (positive control) and naïve mice administered Z03115 (naïve test control). After blocking, no significant fluorescence change was observed for IRDye 700DX in all groups (Figure 2A–G, dashed curves) or for ABY-029 in the vehicle group (Figure 2A,B, solid curves). Surprisingly, postblocking ABY-029 fluorescence exhibited a rise and subsequent decrease over the 60 min range in tumor of positive control and test groups (Figure 2D,F, solid curves) and muscle of positive control, test, and naïve test control groups (Figure 2C,E,G, solid curves).

**Figure 2:**
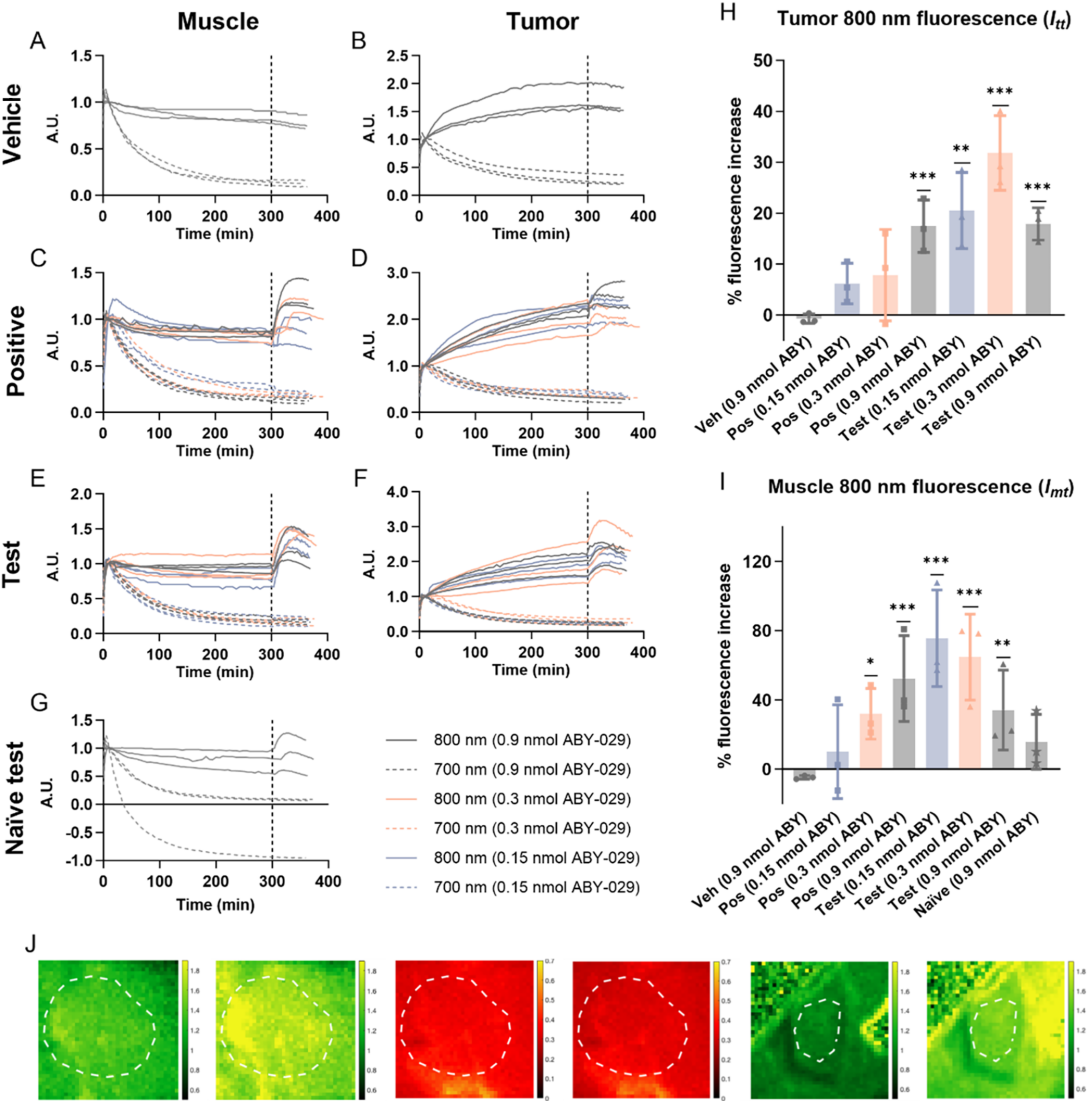
*In vivo* tracking and profiling of paired-agent fluorescence. Kinetic fluorescence curves of ABY-029 (800 nm, solid curve) and IRDye 700DX (700 nm, dashed curve) were plotted separately by animal grouping and tissue type: (A) vehicle muscle, (B) vehicle tumor, (C) positive control muscle, (D) positive control tumor, (E) test muscle, (F) test tumor, and (G) naïve test control muscle. The dashed, vertical lines in (A–G) at 300 min indicate the administration of the blocking agent. (H) Statistically significant increases in tumor ABY-029 fluorescence postreceptor blocking were observed in positive control mice imaged with 0.9 nmol ABY-029 and test mice imaged with all doses of ABY-029. (I) Muscle ABY-029 fluorescence exhibited significant increases by blocking in the test mice imaged with 0.3 or 0.9 nmol ABY-029 and test mice imaged with all doses of ABY-029. Statistical significance in (H, I) was based on Tukey’s test following RM ANOVA. (J) Representative fluorescence images from one test group mouse. Image display: (from left to right) tumor ABY-029 at 270 min, tumor ABY-029 at 330 min, tumor IRDye 700DX at 270 min, tumor IRDye 700DX at 330 min, muscle ABY-029 at 270 min, and muscle ABY-029 at 330 min. The white dashed circle in each image underlines ROI (ABY: ABY-029, AU: arbitrary unit, Pos: positive control, Veh: vehicle control. *p < 0.05, **p < 0.01, ***p < 0.001. Bar: mean ± SD. Dot: individual value).

Pre- and postblocking ABY-029 fluorescence in tumor and muscle are summarized in Table S1. In both tumor and muscle, divergent fluorescence changes postblocking were observed among animal groups. Yet, the unexpected postblocking increase in ABY-029 fluorescence, opposite to preassumption for one-tailed, two-way RM ANOVA, resulted in close-to-one p values: for tumor, *F*(6, 14) = 6.27, *p* = 0.9999; for muscle, *F*(7, 16) = 6.02, *p* = 0.99995. Two-tailed, two-way RM ANOVA was thus reported for reference: for tumor, *F*(6, 14) = 6.27, *p* = 0.002; for muscle, *F*(7, 16) = 6.02, *p* = 0.001. Tukey’s test identified significant increases in ABY-029 fluorescence in tumors of positive control mice imaged with 0.9 nmol of ABY-029 and test mice imaged with 0.15, 0.3, or 0.9 nmol of ABY-029, as well as in the muscle of positive control mice imaged with 0.3 or 0.9 nmol of ABY-029 and test animals imaged with 0.15, 0.3, or 0.9 nmol of ABY-029. Percentage increases in fluorescence postblocking were calculated and summarized in Figure 2H,2I, respectively.

### Characterization and Kinetic Profiling of RA

BP, a value proportional to RA, was calculated for all tumors using both the “deconvolution” approach (BP_deconv_) and the “ratiometric” approach (BP_ratio_). The BP–time curves are summarized in Figure 3 (BP_deconv_) and Figure S1 (BP_ratio_), respectively. Preblocking BP kinetics exhibited similarities among all groups, with an initial increase to a steady-state plateau (Figures 3A–C and S1A–C). Preblocking BP values were calculated from the plateau, which represented the maximal tumor EGFR available for binding (Figures 3E, S1E and Table S2). No statistically significant difference was observed in preblocking BP_deconv_ or BP_ratio_ among animals imaged with different doses of ABY-029 (Figures 3E and S1E).

**Figure 3:**
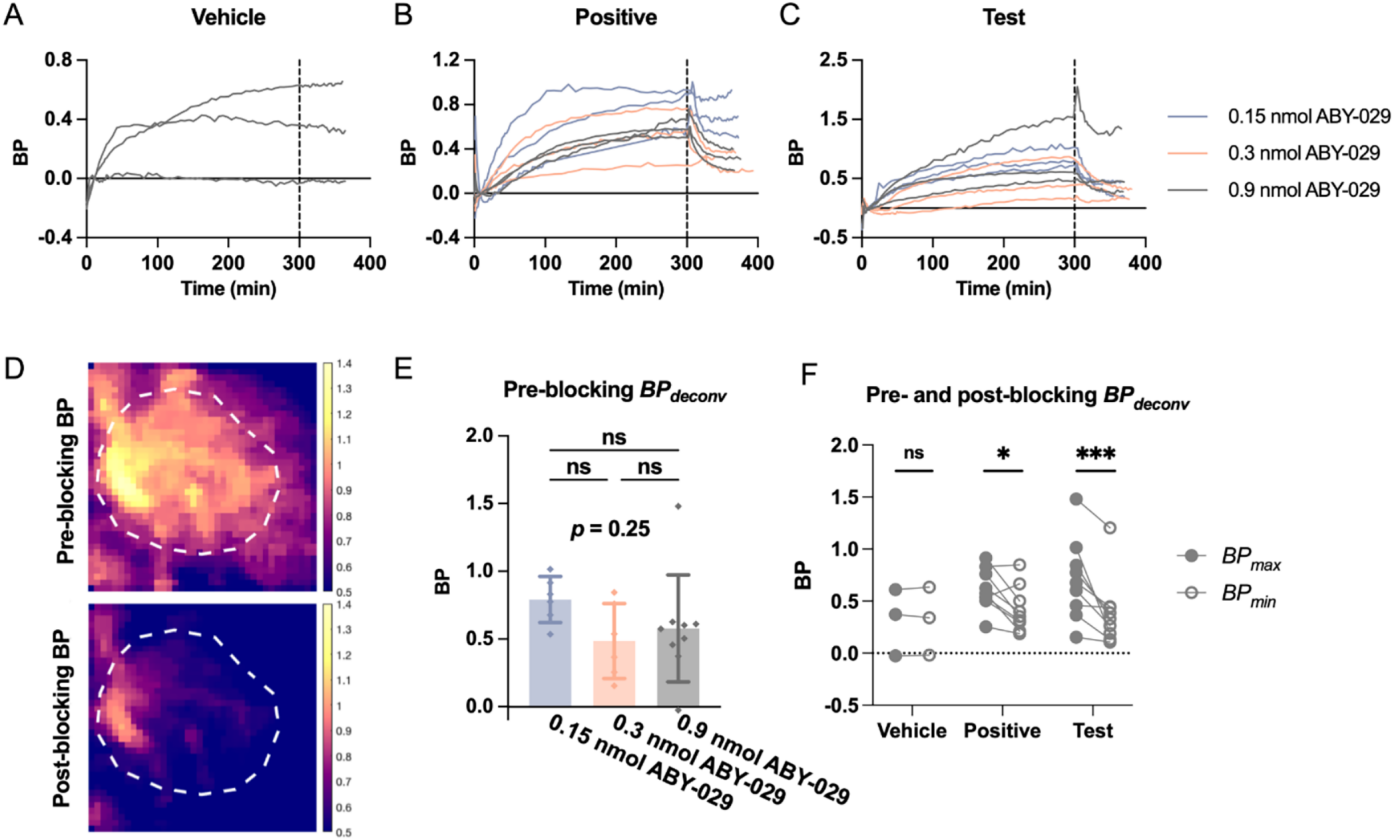
Dynamic tracking and profiling of *in vivo* RA, represented by BP_deconv_. Kinetic BP curves were plotted separately for (A) vehicle, (B) positive control, and (C) test groups. The dashed vertical lines in (A–C) at 300 min indicate blocking agent administration. (D) Representative BP maps from one test group mouse. Image display: (top) BP_deconv_ at 270 min and (bottom) BP_deconv_ at 330 min. The white dashed circle in each image underlines the tumor ROI. (E) No significant differences in preblocking BP_deconv_ were observed among animals imaged with different doses of ABY-029. (F) Statistically significant decreases in tumor BP postblocking were observed in positive control and test group mice. Statistical significance was based on Tukey’s test following one-tailed RM ANOVA. (ABY: ABY-029, BP: binding potential, Pos: positive control, Veh: vehicle control. *p < 0.05, **p < 0.01, ***p < 0.001. Bar: mean ± SD. Dot: individual value)

Both the positive control and test groups exhibited kinetic decreases in postblocking BP (Figures 3B,C and S1B,C), while the vehicle group exhibited minor changes in BP (Figures 3A and S1A). Postblocking BPs for all animal groups were calculated and are summarized in Table S2. Treatment-wise comparisons identified significant differences in changes of BP in response to blocking agent administration at 300 min [one-tailed, two-way RM ANOVA, based on the preassumption that postblocking BP is no less than preblocking BP. For BP_deconv_, *F*(2, 18) = 3.44, *p* = 0.027; for BP_ratio_, *F*(2, 18) = 2.75, *p* = 0.045]. Statistically significant decreases in BP were observed in mice administered hEGF (positive control treatment) and Z03115 (test group), while mice injected with PBS showed no significant change in BP.

Postblocking BPs for all groups were calculated and are summarized in Table S2. Percent decreases in BP were calculated and are summarized in Figure S2A,B. Mice blocked with PBS exhibited a near-zero percent drop in BP, while larger percentage decreases were observed in positive control and test groups (Figure S2A,B). Statistically significant decreases in BP were observed in the positive control mice imaged with 0.9 nmol and test mice imaged with 0.15 or 0.3 nmol of ABY-029 (BP_deconv_, Figure S2A); or in the positive control mice imaged with 0.9 nmol, and test mice imaged with 0.15 or 0.9 nmol of ABY-029 (BP_ratio_, Figure S2B).

## Discussion

An accurate method of measuring drug RA could bring substantial benefits to both drug development and therapeutic assessment in patients by providing insights into dose–response relationships.^3–6^ Previously, we have introduced *in vivo* PAI for measuring RA in a static setting.^30,31,43^ The first study demonstrated that administration of saturating levels of hEGF decreased tumor BP at 1 h and both tumor fluorescence and BP at 24 h postadministration.^30^ A second study using paired-agent fluorescence tomography showed a statistically significant BP decrease in mice administered a single dose of cetuximab 24 h before imaging, compared to untreated mice.^35^ While the potential to measure RA was demonstrated in those studies, PAI has never been tested for dynamic tracking of RA in individuals.

In this study, near-infrared PAI was employed for tumor RA measurement *in vivo*, where both fluorescence and paired-agent BP were dynamically monitored for 6 h to capture imaging agent administration to receptor blocking. Unexpectedly, it was observed that during the postblocking period, ABY-029 fluorescence in both tumor and muscle increased in all animal groups apart from the vehicle control. Our hypothesis for this phenomenon is that receptor competition from excess blocking agents caused EGFR-bound ABY-029 throughout the body (liver, kidney, skin, etc.) to be released back into the blood, increasing measured signal in tumor and muscle. This theory is supported by the effect being observed in the naïve mouse cohort and that ABY-029 binds to both human and rodent EGFR.^39^ Further studies utilizing kinetic modeling are needed for validation. Regardless, this phenomenon invalidated the feasibility of kinetic RA monitoring using a receptor-targeted agent alone.

The PAI method for determining RA performed as anticipated, as BP reports on the concentration of EGFR available for binding.^32^ It also confirms our assumption that administration of trace level ABY-029 (occupying <5% of the total receptor) holds true.^44^ Another inherent assumption of the compartment model for PAI is the adiabatic approximation for tissue homogeneity, which assumes instantaneous mixing of the imaging agents within the tumor “compartment”.^24,45^ If this assumption was true, *in vivo* BP should reach a plateau instantaneously after paired-agent administration and hold constant. This was not observed *in vivo*, where time is required to reach a steady state, and explains the “increase-to-plateau” observed in the BP time curves. After administration of the blocking agent at 300 min, a dynamic decrease in BP was observed in mice administered hEGF (positive control blocking agent) and Z03115 (test group agent), but not the ones given the negative control agent. Hence, it is reasonable to assume that the large concentrations of hEGF and Z03115 administered decreased the concentration of EGFR available for binding (i.e., RA). This time-dependent change in BP demonstrates the potential of PAI to dynamically track changes in tumor RA *in vivo*. Although the trend of BP–time curves was consistent between BP_deconv_ and BP_ratio_, the BP_deconv_-time curves appeared to be more stable, suggesting the superiority of BP_deconv_ for *in vivo* RA assessment.

Group-wise comparisons of pre- vs postblocking ABY-029 fluorescence administered hEGF or Z03115 failed to reach statistical significance, potentially due to the high interindividual variability observed from fluorescence and low sample size (3 mice/group) for each imaging agent dose group. However, for RA assessment using PAI, we showed that preblocking BP was the same for all imaging agent administration doses, demonstrating the utility of this ratiometric technique for standardizing measurements. As such, we were able to analyze each treatment group (hEGF and Z03115) as one large cohort and demonstrated that both treatment groups yielded a statistically significant difference between the pre- and postblocking BP (BP_max_ and BP_min_, respectively). However, when the percent difference from each dose group was analyzed as it was for fluorescence (Supporting Data), we observed similar interindividual variance. It is expected that increasing the sample size per group would lead to more robust statistical analyses. On the other hand, the large variation may also indicate the necessity of performing *in vivo* RA assessment in individuals and reporting based on “per animal” measurements for future studies.

Despite a reported maximum tissue penetration of approximately 3 cm,^46^ the imaging depth limitation of fluorescence imaging may hinder the application of near-infrared PAI in measuring patient RA in deep embedded tumors. However, the strategy of quantitative PAI is not limited to fluorescence molecular imaging. All modalities of molecular imaging, such as SPECT, of which signals from the two simultaneously administered imaging agents can be distinguished and quantified, may have the potential to measure RA using the PAI method. This study represents one of the primary investigations into the application of PAI for RA assessment.

## Conclusions

This study has demonstrated that PAI was capable of dynamically tracking tumor RA (represented by BP) in individual animals. The RA measured by PAI was independent of targeted agent dosage, and receptor blocking was accurately reported by a kinetic decrease in BP. On the other hand, real-time targeted agent fluorescence demonstrated an increase in both tumor and muscle tissues, failing to represent the receptor-blocking process.^30^ The current study did not employ commonly used monoclonal antibodies for treating HNC and a wide range of other EGFR-positive cancer types.^47,48^ In future studies, FDA-approved cetuximab antibody therapy for EGFR inhibition in HNC will be employed to accurately recapitulate HNC therapy. In addition, our previously demonstrated quantitative characterization of skin fluorescence to correct for skin will be utilized,^49^ which will negate the need to surgically expose the time in long-time imaging studies. Taken together, noninvasive dynamic PAI could be a powerful tool for *in vivo* RA tracking in both basic science and translational studies.

## Supplementary Material









## Notes

Dynamic tracking and profiling of *in vivo* receptor availability; represented by binding potential calculated from the ratiometric approach (Supporting Figure 1); percent decrease of *in vivo* receptor availability (RA) represented by binding potential (BP) calculated from the deconvolution and ratiometric approaches (Supporting Figure 2); summary of preblocking and postblocking ABY-029 fluorescence in tumor and muscle (Supporting Table 1); summary of preblocking and postblocking binding potential calculated from the deconvolution and ratiometric approaches (Supporting Table 2) (PDF)
Tumor fluorescence and BP from a representative mouse in the test group (Video 1) (AVI)
Tumor fluorescence and BP from a representative mouse in the vehicle control group (Video 2) (AVI)
Tumor fluorescence and BP from a representative mouse in the positive control group (Video 3) (AVI)

## Abbreviations

BP: binding potential
BRET: bioluminescence resonance energy transfer
CIP: companion imaging probes
EGFR: epidermal growth factor receptor
hEGF: human epidermal growth factor
GLP: good laboratory practices
HNC: head and neck cancer
MFI: mean fluorescence intensity
PAI: paired-agent imaging
PBS: phosphate-buffered saline
PET: positron emission tomography
RA: receptor availability
RO: receptor occupancy
SPECT: single-photon emission computed tomography
