# Supplementary material for: Dynamic Tracking of In Vivo Receptor Availability in Tumor Using Paired-Agent Imaging
Source: Mol Pharm. 2025 May 14;22(6):3142–50. doi: 10.1021/acs.molpharmaceut.5c00060 (PMC12135036; doi:10.1021/acs.molpharmaceut.5c00060)
Supplement: Supplementary file 1 [file mp5c00060_si_001.pdf]

Dynamic tracking of in vivo receptor availability in tumor using paired-agent imaging

Yichen Feng†1, Xiaochun Xu†2, Cody C. Rounds3, Sassan Hodge2, Kenneth M. Tichauer3, and Kimberley S. Samkoe*1,2

[1]Geisel School of Medicine, Dartmouth College, 1 Rope Ferry Road, Hanover, NH 03755, USA
[2]Thayer School of Engineering, Dartmouth College, 15 Thayer Drive, Hanover, NH 03755, USA
[3]Biomedical Engineering, Illinois Institute of Technology, 3255 S Dearborn Street, Chicago, IL 60616, USA

†Contributed equally to this work.

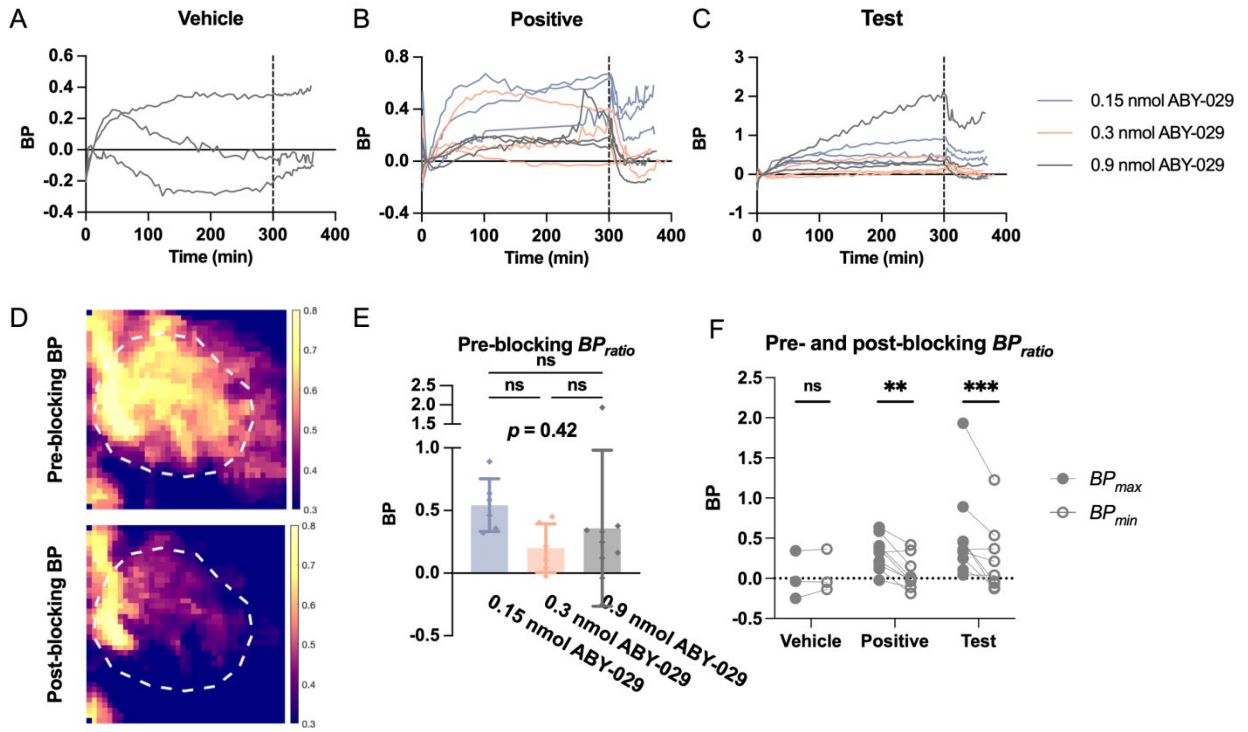

SUPPLEMENTAL FIGURE 1. Dynamic tracking and profiling of *in vivo* receptor availability (RA), represented by binding potential (BP) calculated from the "ratiometric" approach (BP_{ratio}). Kinetic BP curves were plotted separately for: (A) vehicle, (B) positive control, and (C) test groups. The dashed, vertical lines in (A)-(C) at 300 min indicate blocking agent administration. (D) Representative BP maps from one test group mouse. Image display: (top)

BP_{ratio} at 270 min and (bottom) BP_{ratio} at 330 min. The white, dashed circle in each image underlines tumor ROI. (E) No significant difference in pre-blocking BP_{ratio} was observed among animals imaged with different doses of ABY-029 [One-way analysis of variance (ANOVA) with Tukey's test]. (F) Statistically significant decreases in tumor BP post-blocking were observed in positive control and test group mice. Statistical significance in (F) was based on Tukey's test following one-tailed, two-way repeated-measures (RM) ANOVA. [ABY: ABY-029, Pos: positive control, Veh: vehicle control. *$p < 0.05$, **$p < 0.01$, ***$p < 0.001$. Bar: mean ± standard deviation (SD). Dot: individual value]

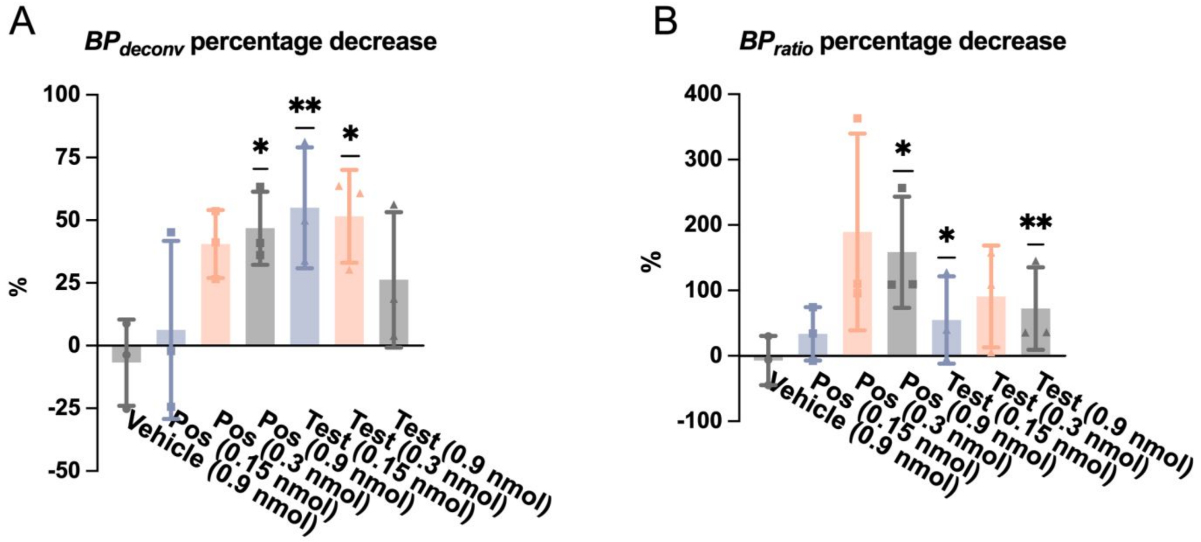

SUPPLEMENTAL FIGURE 2. Percent decrease of *in vivo* receptor availability (RA) represented by binding potential (BP) calculated from the (A) "deconvolution" approach (BP_{deconv}) and (B) "ratiometric" approach (BP_{ratio}). Statistical significance in (F) was based on Tukey's test following one-tailed, two-way repeated-measures (RM) ANOVA. [Test: Z03115 test group, Pos: hEGF positive control, Veh: vehicle control. *$p < 0.05$, **$p < 0.01$, ***$p < 0.001$. Bar: mean ± standard deviation (SD). Dot: individual value]

SUPPLEMENTAL TABLE 1. Summary of pre-blocking and post-blocking ABY-029 fluorescence (800 nm) in tumor (I_{tt}) and muscle (I_{mt}).

Fluorescence (AU)	Vehicle	Pos (0.15 nmol)	Pos (0.3 nmol)	Pos (0.9 nmol)	Test (0.15 nmol)	Test (0.3 nmol)	Test (0.9 nmol)	Naïve test control
$I_{tt}(pre)$	2 ± 0.3	2 ± 0.2	2 ± 0.4	2 ± 0.1	2 ± 0.4	2 ± 0.6	2 ± 0.3	NA
$I_{tt}(post)$	2 ± 0.2	2 ± 0.3	2 ± 0.2	3 ± 0.3	2 ± 0.2	2 ± 0.7	2 ± 0.3	NA
$I_{mt}(pre)$	0.8 ± 0.07	0.8 ± 0.08	0.8 ± 0.05	0.8 ± 0.03	0.8 ± 0.1	0.9 ± 0.2	0.9 ± 0.07	0.8 ± 0.2
$I_{mt}(post)$	0.8 ± 0.07	0.9 ± 0.2	1 ± 0.2	1 ± 0.2	1 ± 0.1	1 ± 0.05	1 ± 0.3	0.9 ± 0.3

[illegible]

SUPPLEMENTAL TABLE 2. Summary of pre-blocking and post-blocking tumor BP calculated from the deconvolution (BP_{deconv}) and ratiometric (BP_{ratio}) approaches.

BP	Vehicle	Pos (0.15 nmol)	Pos (0.3 nmol)	Pos (0.9 nmol)	Test (0.15 nmol)	Test (0.3 nmol)	Test (0.9 nmol)	Naïve test control
$BP_{deconv}(pre)$	0.3 ± 0.3	0.8 ± 0.2	0.5 ± 0.3	0.6 ± 0.06	0.8 ± 0.2	0.5 ± 0.4	0.8 ± 0.6	NA
$BP_{deconv}(post)$	0.3 ± 0.3	0.7 ± 0.2	0.3 ± 0.09	0.3 ± 0.09	0.3 ± 0.1	0.2 ± 0.1	0.6 ± 0.5	NA
$BP_{ratio}(pre)$	0.02 ± 0.3	0.5 ± 0.2	0.2 ± 0.2	0.2 ± 0.1	0.6 ± 0.3	0.2 ± 0.2	0.8 ± 0.9	NA
$BP_{ratio}(post)$	0.06 ± 0.3	0.3 ± 0.1	-0.04 ± 0.07	-0.08 ± 0.1	0.3 ± 0.3	-0.02 ± 0.05	0.4 ± 0.7	NA

[illegible]

SUPPLEMENTAL VIDEO 1. Example video of tumor fluorescence and BP from a representative mouse in the test group (imaged with 0.9 nmol ABY-029). Left: kinetic map and curve of ABY-029 tumor fluorescence, middle: kinetic map and curve of IRDye 700DX tumor fluorescence and right: kinetic map and curve of tumor BP_{deconv} (unitless).

SUPPLEMENTAL VIDEO 2. Example video of tumor fluorescence and BP from a representative mouse in the vehicle control group (imaged with 0.9 nmol ABY-029). Left: kinetic map and curve of ABY-029 tumor fluorescence, middle: kinetic map and curve of IRDye 700DX tumor fluorescence and right: kinetic map and curve of tumor BP_{deconv}.

SUPPLEMENTAL VIDEO 3. Example video of tumor fluorescence and BP from a representative mouse in the positive control group (imaged with 0.9 nmol ABY-029). Left: kinetic map and curve of ABY-029 tumor fluorescence, middle: kinetic map and curve of IRDye 700DX tumor fluorescence and right: kinetic map and curve of tumor BP_{deconv}.
